# Assessment of bedside lower limb angiography combined with continuous NIRS monitoring for the detection of lower limb complications of VA-ECMO: an observational monocentric study

**DOI:** 10.1186/s13054-021-03703-5

**Published:** 2021-07-31

**Authors:** Matthieu Koszutski, Mathieu Mattei, Juan Pablo Maureira, Antoine Kimmoun, Bruno Levy

**Affiliations:** 1 CHRU de Nancy, Médecine Intensive et Réanimation Brabois, Université de Lorraine, Nancy, France; 2 CHRU de Nancy, Service de Chirurgie Cardiaque, Université de Lorraine, Nancy, France; 3 CHRU de Nancy, Médecine Intensive et Réanimation Brabois, INSERM U1116, Université de Lorraine, Nancy, France

## Abstract

*Trial registration*: ClinicalTrials.gov Identifier: NCT03910062.

**Dear Editor,**

Peripheral VA-ECMO is used as a temporary cardiac support in stage D and E of cardiogenic shock [1]. To prevent lower limb ischemia due to the occlusive effect of the arterial cannula in the femoral artery, a limb reperfusion cannula is usually placed in the ipsilateral superficial femoral artery [2]. A few published case reports suggest that angiography through the reperfusion cannula is efficient to detect and monitor the treatment of the arterial adverse events during VA-ECMO [3, 4]. This procedure has never been correctly formalized and evaluated.

This monocentric study is a prospective evaluation of a strategy to prevent lower limb complications during VA-ECMO with a systematic arterial angiography through the reperfusion line, on VA-ECMO implantation and when limb ischemia is suspected, in addition to continuous lower-limb near infrared spectroscopy (NIRS) monitoring (https://clinicaltrials.gov/ct2/show/NCT03910062). When performed in the ICU, a digital mobile X-ray device was used with the panel sensor placed underneath the patient's leg to ensure the of visualization of both the superficial femoral and the popliteal arteries. A 10 ml to 20 ml dose of iodinated contrast was injected through the three-way stopcock towards the reperfusion cannula, followed by the X-ray acquisition. Lower limb tissue oxygenation was continuously monitored by NIRS (Masimo-Root® with O3®-Regional Oximetry). Limb ischemia was defined as a NIRS value < 50% on the ipsilateral limb and/or a differential between lower limbs >15% [5]. The primary outcome was the incidence of severe lower limb ischemia (ischemia leading to a surgical intervention, functional sequelae, extremities necrosis or compartment syndrome) at day 60.

From June 2019 to April 2020, 39 consecutive patients were included at Nancy's Teaching Hospital. Patients’ characteristics and outcomes are reported in Table 1. Indication for VA-ECMO was refractory cardiogenic shock (*n* = 14) or refractory cardiac arrest (*n* = 25). Eleven (28%) patients were cannulated with a percutaneous ultrasound-guided technique and 28 (72%) by surgical cut-down. The high rate of refractory cardiac arrest explains the high rate of surgical cut-downs as the surgical technique being preferred at our center for extracorporeal cardiopulmonary resuscitation. The reperfusion cannula was a 6F (AVANTI® + , Cordis, USA) sheath introducer (ultrasound-guided percutaneous insertion or under visual control in case of surgical cut-down).

**Table 1:** Characteristics of the study population and outcomes

Demographic characteristics	*n* = 39
Age, years	58 (46, 64)
Male sex, *n* (%)	30 (77%)
BMI (kg/m^2^)	28 (24, 29)
Simplified acute physiological score 2	74 (56, 92)
SOFA score at admission	13 (10, 14)
Comorbidities, *n* (%)	
Chronic kidney failure	2 (5%)
Diabetes	5 (13%)
Occlusive peripheral arterial disease	4 (10%)
Long-term antiplatelet therapy	8 (21%)
History of smoking > 10 pack-years	17 (44%)
VA-ECMO indication	
E-CPR (OHCA and IHCA)	24 (62%)
Refractory Cardiogenic Shock	15 (38%)
Canulation technique	
Percutaneous canulation	11 (28%)
Surgical approach	28 (72%)
Primary outcome	
Severe lower limb ischemia (amputation, fasciotomy or surgical intervention)	1 (3%)
Secondary outcomes	
Lower limb ischemia (NIRS < 50% and /or differential > 15%)	9 (23%)
ICU mortality, *n* (%)	20 (51%)
Duration of ICU stay, days	10 (5, 18)
Duration of hospital stay, days	17 (7, 42)
Renal replacement therapy during the ICU stay	15 (39%)

Data are presented as the median (Q1, Q3) or number of patients with the percentage in parentheses, as appropriate

*BMI* body mass index, *SOFA score* sequential organ failure assessment score, *OHCA* out-of-hospital cardiac arrest, *IHCA* in-hospital cardiac arrest

Forty-three angiographies were performed during the study period. Thirty-seven angiographies were performed at VA-ECMO implantation (not performed = 2). Six adverse events related to the reperfusion cannula were detected at VA-ECMO implantation: four misplacements (in the deep femoral artery or the femoral vein), one reperfusion cannula kinking and one hemorrhage secondary to an arterial puncture (Fig. 1). The detection of reperfusion cannula abnormalities at VA-ECMO implantation allowed a prompt intervention without subsequent limb ischemia. The four misplacements occurred with the surgical technique under high doses of norepinephrine.

**Fig. 1:**
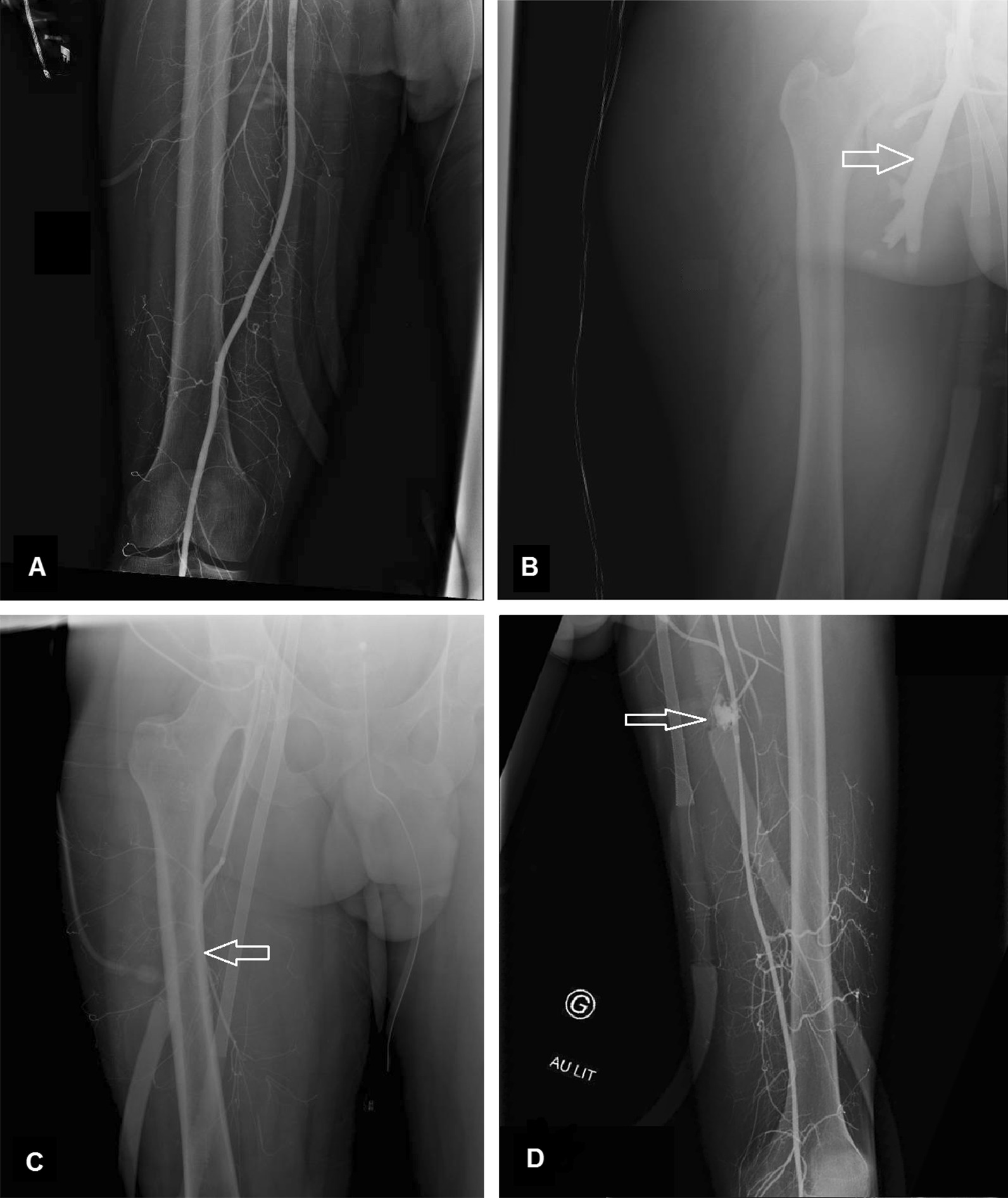
Common findings on the bedside-angiography after VA-ECMO implantation. **a** Normal angiography showing an opacification of the superficial femoral and popliteal arteries. **b** Angiography of a reperfusion cannula misplaced in the deep femoral vein. **c** Reperfusion cannula misplaced in the deep femoral artery. **d** An active hemorrhage following a previous failed attempt of placing the reperfusion cannula

During the run of VA-ECMO, nine ischemic events were suspected based on NIRS criteria. The cause for limb ischemia was obvious in three cases (e.g. reperfusion cannula thrombosis) and a bedside angiography was performed for the six remaining. The angiographic findings were: two thrombosis, one vasospasm, one with both and two normal. One patient (arterial thrombosis on angiography) ultimately required an amputation. The remaining ischemic events were all resolutive with a medical treatment (e.g. vasodilators, ECMO flow increase). There was no complication recorded due to the angiography procedure.

In conclusion, bedside angiography through the lower limb reperfusion is a safe and efficient procedure in the management of arterial complications during VA-ECMO. Combined with continuous NIRS monitoring, this technique allows a fast diagnosis of vasospasm or thrombosis when ischemia is suspected. Performed at VA-ECMO implantation, it confirms that the reperfusion cannula is properly positioned in the superficial femoral artery.

## Data Availability

The datasets used and/or analysed during the current study are available from the corresponding author on reasonable request.
